# Molybdenum Nitride and Oxide Layers Grown on Mo Foil for Supercapacitors

**DOI:** 10.3390/ma18245649

**Published:** 2025-12-16

**Authors:** Dong Hyun Lim, Young-Il Kim

**Affiliations:** Department of Chemistry, Yeungnam University, 280 Daehak-ro, Gyeongsan 38541, Republic of Korea; dong9535@naver.com

**Keywords:** molybdenum nitrides, molybdenum dioxide, supercapacitor, binder-free electrode

## Abstract

In this study, thin molybdenum nitride (MoN*_x_*) layers were directly synthesized on molybdenum foil via thermal treatment under an NH_3_ atmosphere, and their phase evolution, structural characteristics, and electrochemical performance were investigated. The thickness and morphology of the MoN*_x_* layers were controlled by varying ammonolysis time and temperature, while subsequent annealing in N_2_ converted the nitride layer into MoO_2_. Meanwhile, oxidation in air yielded crystalline MoO_3_ layers. X-ray diffraction and X-ray photoelectron spectroscopy confirmed progressive oxidation of molybdenum, with Mo 3*d* binding energies increasing in the sequence of Mo < MoN*_x_* < MoO_2_ < MoO_3_, consistent with their nominal oxidation states. Electrochemical characterization revealed that both MoN*_x_*/Mo and MoO_2_/Mo electrodes exhibit notable pseudocapacitive behavior in 0.5 M H_2_SO_4_ electrolyte, with areal specific capacitances reaching up to 520 mF cm^−2^ at 10 mV s^−1^. Increasing layer thickness led to enhanced capacitance, likely due to an increase in the electrochemically accessible surface area and the extension of ion diffusion pathways. MoO_2_-coated samples showed stronger faradaic contribution and superior rate capability compared to MoN*_x_* counterparts, along with a gradual shift from predominantly electric double-layer capacitance toward hybrid pseudocapacitive charge storage mechanisms.

## 1. Introduction

With increasing global concern regarding energy sustainability and environmental impact, there is a growing demand for advanced technologies that enhance energy efficiency and power management. Energy storage plays a central role in modern power systems and is becoming increasingly important due to the rapid expansion of renewable energy sources [1,2]. Energy storage and conversion are realized through various device types such as rechargeable batteries, supercapacitors, and fuel cells, each distinguished by their working mechanisms and power/energy densities [3,4,5]. Supercapacitors represent a relatively recent energy storage strategy that offers high power delivery, rapid charge–discharge rates, and long cycle life [6,7,8]. However, their energy density generally remains lower than that of batteries. The electrochemical performance of supercapacitor electrodes can be significantly tuned not only by the selection of electroactive materials but also by the fabrication strategy employed. To date, promising supercapacitor electrodes have been developed predominantly using carbon-based materials (activated carbon, graphene, and nanotubes) [9,10,11,12] or transition metal-based compounds (oxides [13], oxynitrides [14,15], and sulfides [16]). Numerous studies therein have emphasized controlling the morphology to increase the specific surface area and maximize active sites for charge transfer [17,18,19]. At the same time, the adhesion quality between the active material and current collector has been identified as a critical factor affecting charge transport kinetics and cycling stability. Slurry-based drop casting is perhaps the most widely adopted approach due to its simplicity [20,21], but it often suffers from poor interfacial adhesion and mechanical instability. To overcome these drawbacks, thin film deposition methods such as electrodeposition [22,23,24], sputtering [25,26,27], chemical vapor deposition [28,29], and atomic layer deposition [30] have been employed, although these deposition techniques typically require sophisticated equipment and multistep processing. In this regard, the development of a simple, scalable, and robust strategy of fabricating binder-free supercapacitor electrodes is highly desirable.

Recently, we reported on the surface modification of titanium foil through ammonolytic nitridation, producing TiN/Ti structures with promising electrochemical performance [31]. This nitridation strategy is believed to be applicable to other transition metals. Among them, molybdenum (Mo) is a particularly attractive candidate due to its wide range of nitrides and oxides, as well as its diverse redox behavior. Molybdenum and nitrogen form several line compounds including β-Mo_2_N (tetragonal) [32], γ-Mo_2_N (cubic) [33], δ-MoN (hexagonal) [34], Mo_5_N_6_ (hexagonal) [34], and Mo_2_N_3_ (cubic) [35]. The cubic/tetragonal phases (β-Mo_2_N, γ-Mo_2_N, and Mo_2_N_3_) have a rock salt-type atomic arrangement, δ-MoN has a NiAs-like hexagonal lattice, and Mo_5_N_6_ is an intergrowth of WC and NiAs-type structures. The above structures tolerate high densities of atomic vacancies, favoring the formation of non-stoichiometric MoN*_x_* phases. This behavior is further facilitated by the strong covalency of Mo–N and Mo–Mo bonds along with the ability of Mo to adopt diverse oxidation states. Among the various molybdenum nitride phases, hexagonal MoN and cubic Mo_2_N have been frequently reported as promising electrode materials for supercapacitors owing to their high electrical conductivity and chemical stability in acidic electrolytes.

Molybdenum compounds such as MoN, Mo_2_N, MoO_2_, and MoO_3_ have been actively explored for supercapacitors [36,37,38,39,40], batteries [41,42], and electrocatalysts [43,44,45,46]. However, most studies rely on powder synthesis followed by slurry processing, which limits interfacial integrity. To achieve improved performances, however, more sophisticated techniques [47] were also exploited, such as hydrothermal loading of oxide precursor followed by ammonolysis [48], electrodeposition of oxide followed by ammonolysis [49], electrodeposition of MoO_2_ [50], and microwave irradiation for MoO_2_ [51]. Particularly, a recent study prepared a MoN*_x_*@Mo structure by thermal ammonolysis of Mo foil [39]. Therefore, investigating phase evolution and electrochemical behavior in directly transformed dense Mo foil offers an opportunity to develop mechanically robust and functionally efficient electrode architectures. In addition, it is intriguing to investigate the preferred phases that form when a dense Mo foil undergoes thermal treatment in different atmospheres. In this work, we investigate the thermal modifications of Mo foil in NH_3_, N_2_, and air atmospheres and evaluate the resulting phases using X-ray diffraction (XRD), X-ray photoelectron spectroscopy (XPS), and electrochemical methods. The relationship between phase composition, microstructure, and charge storage mechanism is elucidated, providing insight into capacitive behavior of Mo-based coatings.

## 2. Materials and Methods

Molybdenum foil was used as the starting material for synthesizing molybdenum nitrides and oxides. As-purchased Mo foil (Alfa Aesar (Ward Hill, MA, USA), 99.95%, 0.127 mm thick) was cut into 1.5 cm × 1.5 cm square pieces, immersed in 1 N HNO_3_(*aq*) for 5 min, rinsed with deionized water, and dried under N_2_ at 50 °C. Then, the Mo foil was placed inside an alumina tube and heated in NH_3_ flow (PSG Co. (Pusan, Republic of Korea), 99.9999%), N_2_ flow (Shumachemia (Yeongcheon, Republic of Korea), 99.999%), or static air. Particularly for ammonolytic nitridation, the alumina tube was purged with flowing NH_3_ for 15 min to remove residual air. Then, the furnace temperature was raised to the target temperature at a ramp rate of 10 °C min^−1^, and held for a designated reaction time. After completion of the ammonolysis, the furnace was allowed to cool naturally to room temperature. The NH_3_ flow was maintained at a constant rate during the entire heat treatment procedure.

The powder XRD pattern was collected in the Bragg–Brentano mode using a diffractometer (MiniFlex 600, Rigaku (Akishima, Japan)) with Cu *K*_α_ radiation generated at 40 kV, 15 mA. For select samples, synchrotron XRD was also measured in the high-resolution diffractometer installed at the beamline 9B of Pohang Accelerator Laboratory (PAL, Pohang, Republic of Korea), where parallel beam radiation with λ = 1.5219 Å was supplied from the storage ring (3.0 GeV, 250 mA). The diffraction pattern was collected in the Bragg–Brentano mode at a 2θ step of 0.01° and with a scan time of 1 s per step. The lattice constants of molybdenum nitride and oxide phases were refined by the Le Bail fitting [52] of the synchrotron XRD pattern, using the GSAS-I software suite [53,54].

The plane and fracture surfaces of the foil samples were examined using scanning electron microscopy (SEM; S-4200, Hitachi (Tokyo, Japan)). Ultraviolet–visible (UV–Vis) absorption spectra were obtained in a diffuse–reflectance spectrometer (Neosys 2000, Scinco (Seoul, Republic of Korea)) equipped with a 35 mm integrating sphere, using BaSO_4_ as a reference. The pseudo-absorbance (*F*) was retrieved from the diffuse–reflectance (*R*) following the Kubelka–Munk transformation, *F* = (1 − *R*)^2^/2*R* [55]. XPS was performed in a Thermo Scientific (Waltham, MA, USA) K-Alpha spectrometer using Al *K*_α_ source (*h*ν = 1486.6 eV). The signal intensity was recorded at 0.1 eV intervals, and each spectrum was obtained by averaging the results of 20 scans. The XPS binding energy was calibrated using the C 1*s* peak at 284.6 eV, which originated from adventitious carbon.

The electrochemical properties of coated specimens were tested in a potentiostat/galvanostat (VersaSTAT 3, AMETEK (Oak Ridge, TN, USA)) using a three-electrode system employing 0.5 M H_2_SO_4_(*aq*) as an electrolyte. Molybdenum nitride/oxide-coated foil, Pt coil, and Ag/AgCl junction were used as the working, counter, and reference electrodes, respectively. The working electrode was constructed using a custom-made sample holder that exposed only one side of the coated foil (area 0.88 cm^2^). The cyclic voltammetry (CV) was carried out in the potential (*E*) range of −0.2 to +0.5 V vs. Ag/AgCl, at scan rates (v) of 10 to 200 mV s^−1^. The areal specific capacitance, *C*_s_ in F cm^−2^, was calculated as $\int jdE/\left(v\times \Delta E\times A\right)$ from CV, where j, ΔE, and A are areal current density, potential window width, and electrode area, respectively [56]. The galvanostatic charge–discharge (GCD) was conducted in the potential window between −0.1 and +0.5 V vs. Ag/AgCl, and a current density of 0.1 mA cm^−2^.

## 3. Results

### 3.1. Surface Modification of Mo Foil by Heat Treatment

**Figure 1:**
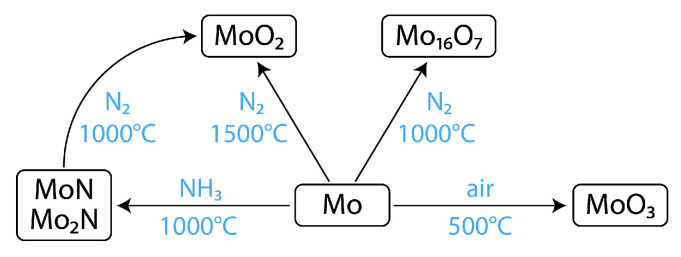
Transformation of molybdenum foil to molybdenum nitrides and oxides.

**Figure 2:**
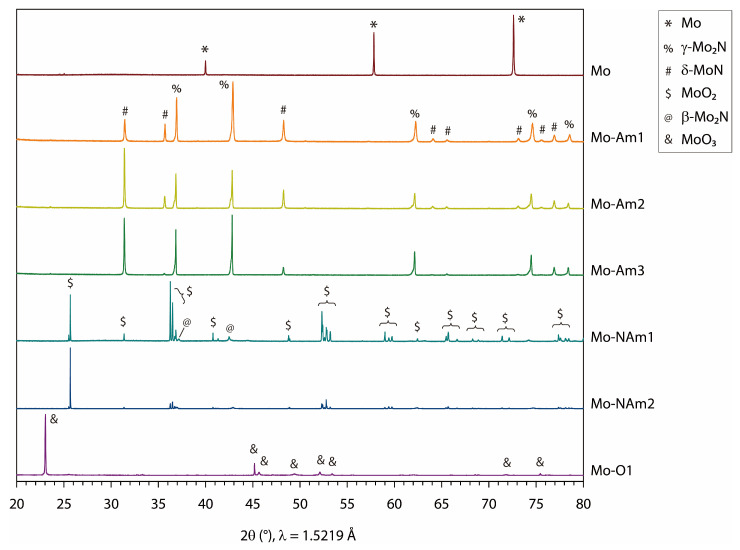
Synchrotron XRD patterns of Mo foil and Mo-derived coatings.

The thermal evolution of Mo foil was investigated in different atmospheres, that is, NH_3_, N_2_, and air, as briefly outlined in Figure 1. A list of representative samples is given in Table 1, and the corresponding synchrotron XRD patterns are presented in Figure 2.

**Figure 3:**
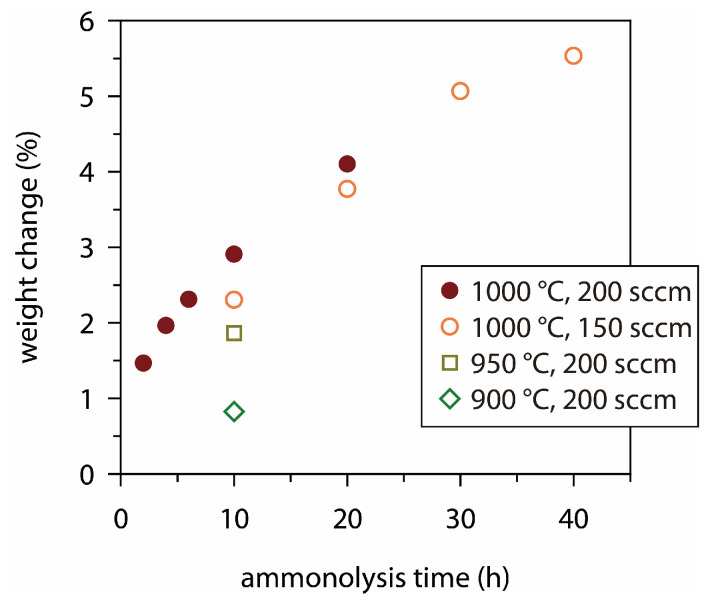
Weight change (Δwt) in Mo foil upon the ammonolytic nitridation, depending on temperature, time, and flow rate of NH_3_.

First, the thermal ammonolysis was carried out using various temperature (*T*_am_ = 900–1000 °C) and time (*t*_am_ = 2–40 h) conditions while keeping the NH_3_ flow rate at 200 mL min^−1^. The ammonolysis resulted in molybdenum nitrides, where the extent of nitridation is directly reflected in the weight change (Δwt) in the foil during heat treatment. For example, a transformation of Mo (FW 95.94) to MoN (FW 109.95) corresponds to a Δwt of +14.6%. Figure 3 shows the experimental Δwt values, indicating that the more vigorous condition leads to the higher nitridation level. With *T*_am_ = 1000 °C and *t*_am_ = 40 h, Δwt was observed to be 5.9%, corresponding to an atomic ratio of N/Mo ≈ 0.4, while with *T*_am_ = 1000 °C and *t*_am_ = 2 h, Δwt was 1.4% (N/Mo ≈ 0.1). The above N/Mo ratios regard the entire foil specimen; therefore, the coated nitride layer will have a higher nitrogen content. As found by the XRD measurement, reactions with *T*_am_ ≤ 900 °C produced a mixture of various nitrides, while the ammonolysis at 1000 °C yielded better-defined products, composed primarily of δ-MoN and γ-Mo_2_N. Previously, bulk forms of molybdenum nitride have been synthesized by various routes, including ammonolysis of Mo powder [57] or MoCl_5_ [58,59], high-pressure and high-temperature reaction between MoO_2_ and NH_4_Cl [60], reduction-nitridation of (NH_4_)_6_Mo_7_O_24_∙4H_2_O-derived MoO_3_ under an N_2_/H_2_ mixture [61], and reaction between Na_2_MoO_4_∙2H_2_O and dicyanamide [35]. Most of these reactions led to a single-phase product of δ-MoN [34], γ-Mo_2_N [33], Mo_2_N_3_ [34], or Mo_5_N_6_ [33]. In several other studies, however, ammonolysis of Mo foil [39] or Mo wire [62] resulted in a mixture of various Mo-N phases. In the present study, we similarly obtained mixed phases of molybdenum nitrides through the ammonolysis of Mo foil. We speculate that the dense texture of the foil or wire impedes the diffusion of the nitriding molecules, leading to a mixture of kinetically accessible phases. The ammonolytic heating of Mo foil at 1000 °C produced two-component mixtures of δ-MoN and γ-Mo_2_N (Figure 2). Judging from the XRD intensity, the phase fractions of the two were similar for Mo-Am1 (*t*_am_ = 2 h), Mo-Am2 (*t*_am_ = 10 h), and Mo-Am3 (*t*_am_ = 20 h). For the cubic γ-Mo_2_N, the relative peak intensities were not influenced by *t*_am_, but for the hexagonal δ-MoN, (00*l*) peaks were enhanced with an increase in *t*_am_ (see Figure S1).

Second, the thermal evolution of Mo foil was examined in N_2_. Heating at 900 °C or below resulted in a poorly crystalline mixture of δ-MoN and γ-Mo_2_N. With the heating at 1000 °C in N_2_, the Mo foil was converted almost exclusively to Mo_16_N_7_ (sample Mo-N1) which is closely related with the γ-Mo_2_N phase [63]. With the heating at 1500 °C in N_2_, however, the Mo foil was converted to MoO_2_ (Mo-N2). The N_2_ gas used in this study was supplied with a nominal purity of 99.999% without additional purification. Nevertheless, possible oxygen sources during high-temperature treatment include residual oxygen or adsorbed species in the alumina tube, trace O_2_ impurities in the gas stream, and minute air leakage under prolonged heating at 1500 °C. These factors are believed to have contributed collectively to the formation of the MoO_2_ phase. The results of Mo-N1 and Mo-N2 indicate that the reactivity of O_2_ is much more dominant at 1500 °C than at 1000 °C. We also examined the modification of MoN*_x_* layers (Mo-Am2 and Mo-Am3) by a subsequent heating in N_2_, and observed the transformation to MoO_2_. Upon heating in the air, the surface of Mo foil was fully oxidized to MoO_3_, but that occurred only at below 600 °C. At higher temperatures such as 900 °C, sample specimens completely sublimed manifesting the volatility of MoO_3_. The synchrotron XRD data (Figure 2) was analyzed by the Le Bail refinement (Figure S2), in which the lattice constants of the coated nitrides and oxides agreed well with the literature data for the bulk phase (Table 2).

### 3.2. SEM, UV-Vis, and XPS

**Figure 4:**
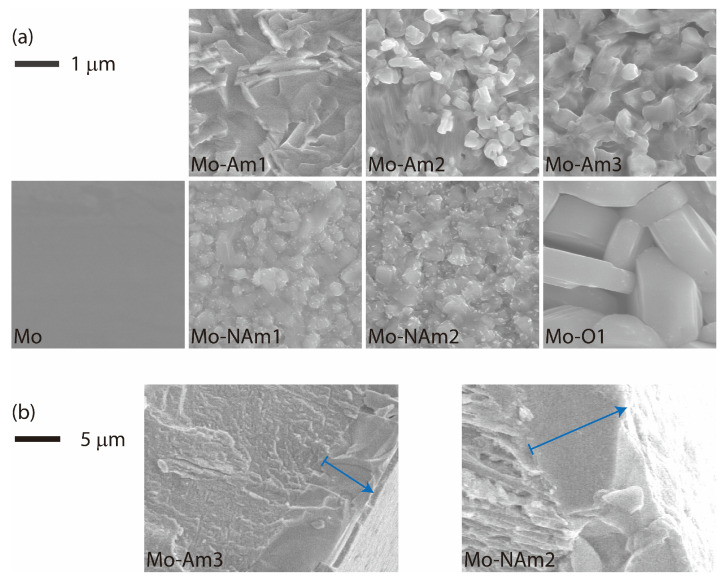
SEM images for (**a**) plane and (**b**) cross-sectional views of Mo foil and Mo-derived coatings. Arrows in (**b**) indicate the coated layers.

The morphology of the Mo-based coatings was examined by SEM. Figure 4 presents the top-view and cross-sectional micrographs of MoN*_x_* (Mo-Am), MoO_2_ (Mo-NAm), and MoO_3_ (Mo-O) layers. The raw Mo foil had a flat and smooth surface, but its nitride and oxide derivatives formed with distinct textures. The MoN*_x_* layers had both platelets and granular particles, presumably associated with hexagonal MoN and cubic Mo_2_N, respectively. The MoO_3_ layer contained large and angulate particles which had edge lengths of several micrometers, indicating a well-crystallized state. The MoO_2_ coatings contained relatively smaller grains with sharp vertices. Comparing between MoN*_x_* and MoO_2_, the former had larger grains along with higher porosity which can provide greater surface area for electrode activity. On the other hand, the smaller and particulate microstructure of MoO_2_ may provide more active sites for pseudocapacitive reactions.

The thickness of the coated layer increased with the time and temperature of heat treatment. In particular, Mo-Am3 and Mo-NAm2 (Figure 4b) had thicknesses of ≈12 μm and ≈18 μm, respectively. Above two coatings were prepared through somewhat similar paths. Compared with Mo-Am3, which was prepared by ammonolysis at 1000 °C for 20 h, Mo-NAm2 was obtained via an additional heat treatment in N_2_. The conversion of MoN*_x_* (Mo-Am3) to MoO_2_ (Mo-NAm2) was accompanied by a substantial volume expansion, which is consistent with the lattice volumes of γ-Mo_2_N (18.0 Å^3^ per Mo), δ-MoN (20.0 Å^3^ per Mo), and MoO_2_ (32.9 Å^3^ per Mo). Such structural expansion may contribute to the formation of additional electrochemically active sites and enhanced electrolyte accessibility within the coating layer.

**Figure 5:**
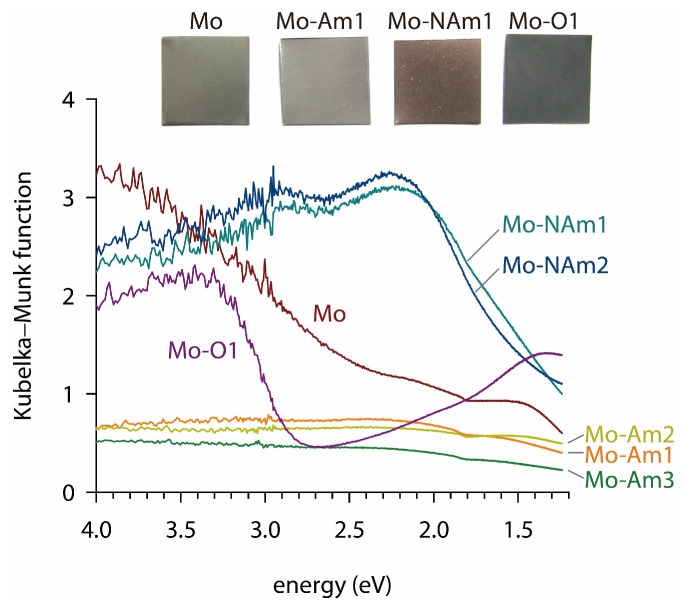
UV-Vis spectra and photograph images of Mo foil and Mo-derived coatings.

Figure 5 presents the UV-Vis spectra and photographic images of Mo foil and Mo-derived coatings. The pristine Mo foil, MoN*_x_* coating (Mo-Am1), and MoO_2_ coating (MoNAm1) exhibited significant absorption over the entire wavelength range of measurement (220–1000 nm), which is characteristic of a metallic or metallic-like electronic structures. The molybdenum in MoN*_x_* (*x* ≤ 1) has an oxidation number lower than 3, and can readily form metallic-like covalent bonding. Similarly, the MoO_2_ coating contains Mo^4+^ (4*d*^2^), and exhibited metallic-like optical characteristics, as established for the bulk MoO_2_ [65]. It is expected that the metallic-like electronic structures of MoN*_x_* and MoO_2_ are accompanied by high electrical conductivity, which is particularly beneficial for achieving enhanced rate capability and efficient charge storage in the supercapacitor electrodes. In contrast, the trioxide MoO_3_ (Mo-O1) with Mo^6+^ (4*d*^0^), was found to be semiconducting with an estimated band gap (*E*_g_) of 2.8 eV. This value agrees well with that of bulk MoO_3_ [65]. The relatively dark appearance of the MoO_3_-coated sample (Mo-O1) is attributed to enhanced optical scattering from its thick and highly crystalline surface with micrometer-scale grains, despite its intrinsically semiconducting nature.

**Figure 6:**
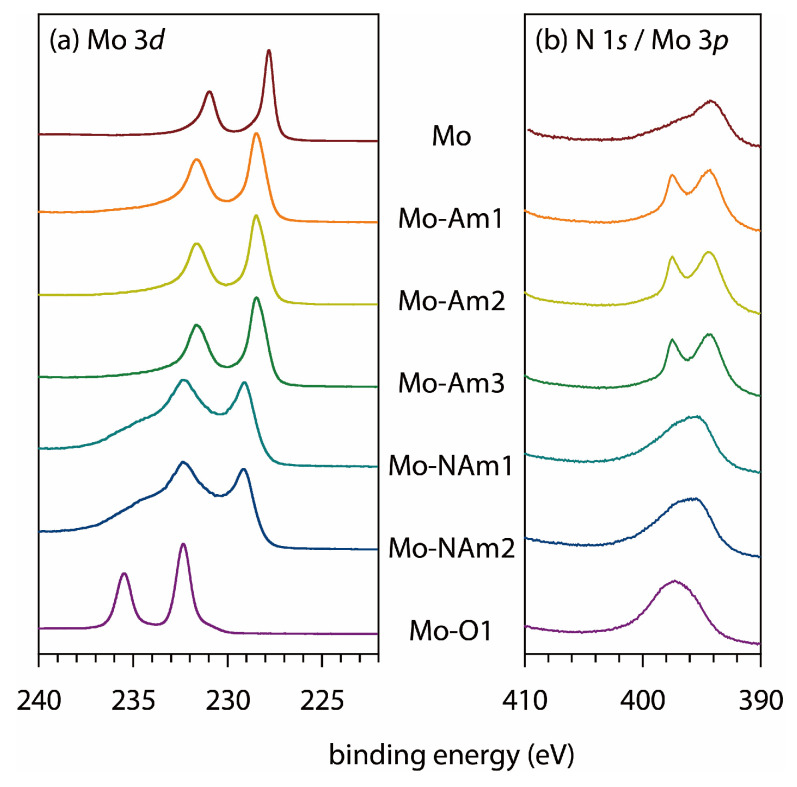
XPS profiles of Mo foil and Mo-derived coatings at the (**a**) Mo 3*d* and (**b**) N 1*s*/Mo 3*p* levels.

The core level XPS data were acquired at Mo 3*d* and N 1*s* regions (Figure 6). The binding energy of Mo 3*d* increased in an order, Mo < Mo-Am < Mo-NAm < Mo-O, in good agreement with the expected variations in the Mo oxidation number [66]. Comparing the two end-compositions, Mo and MoO_3_, both the Mo 3*d*_3/2_ and 3*d*_5/2_ peaks were separated by about 4 eV. The three MoN*_x_* samples (Mo-Am1, Mo-Am2, Mo-Am3) displayed similar spectra in terms of the peak position, and so did the two MoO_2_ samples (Mo-NAm1, Mo-NAm2). Therefore, the Mo 3*d* XPS corroborates the interpretation of the XRD analysis given above.

The N 1*s* and Mo 3*p* levels have similar binding energies to each other, as can be seen in Figure 6b. The N 1*s* line was clearly recognizable from the three MoN*_x_* samples but not from the others. The Mo 3*p* lines did not split clearly to 3*p*_1/2_ and 3*p*_3/2_ components; instead, they occurred as a broad asymmetric profile. The variation in the Mo 3*p* binding energy followed a similar trend to that of the Mo 3*d* line.

### 3.3. Electrochemical Analysis

**Figure 7:**
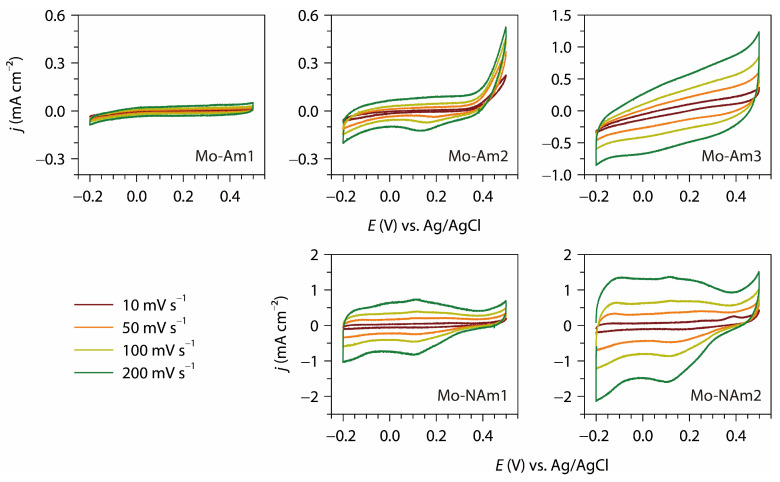
Cyclic voltammograms of MoN_*x*_ (Mo-Am1, Mo-Am2, Mo-Am3) and MoO_2_ (Mo-NAm1, Mo-NAm2) coatings, measured in the potential window of −0.2 to 0.5 V (vs. Ag/AgCl) at scan rates of 10–200 mV s^−1^.

Figure 7 shows the CV diagrams of MoN*_x_* (Mo-Am1, Mo-Am2, Mo-Am3) and MoO_2_ (Mo-NAm1, Mo-NAm2) coatings on the Mo substrate, measured at scan rates ranging from 10 to 200 mV s^−1^. Among the three MoN*_x_* samples, the CV area increased accordantly with the ammonolysis time and thus the thickness of MoN*_x_* layer. This suggests that the coated layers permit electrolyte penetration, allowing internal surfaces to participate effectively in charge storage. A similar trend was observed from the MoO_2_ coatings, where the thicker layer led to the greater redox capacity. The shape of CV profiles reveals differences in the electrochemical behaviors. Mo-Am1 exhibits nearly rectangular and symmetric CV curves with very low current density and the absence of distinct redox peaks, indicating a typical electric double-layer capacitive (EDLC) behavior dominated by physical ion adsorption at the electrode/electrolyte interface. The weak current response suggests a limited electroactive surface area and poor charge storage capability. In contrast, Mo-Am2 and Mo-Am3 show progressively higher current densities accompanied by the appearance of broad humps in the CV curves, which indicates the involvement of faradaic redox reactions in addition to EDLC behavior. The occurrence of these redox features suggests the contribution of surface-controlled pseudocapacitance associated with reversible redox transitions of Mo species, that is, switching between Mo^2+^/Mo^3+^ ↔ Mo^4+^ (in MoN*_x_*) or Mo^4+^ ↔ Mo^5+^/Mo^6+^ (in MoO_2_). Notably, the suboxide samples (Mo-NAm1 and Mo-NAm2) exhibit markedly enhanced current responses and more pronounced redox characteristics. This improvement can be attributed to high electrical conductivity and the introduction of additional active sites, which facilitate rapid surface redox reactions. Among all samples, Mo-NAm2 demonstrates the largest CV area and the most distinct deviation from an ideal rectangular shape, indicating a hybrid charge storage mechanism involving both surface pseudocapacitive behavior and diffusion-controlled faradaic processes.

**Figure 8:**
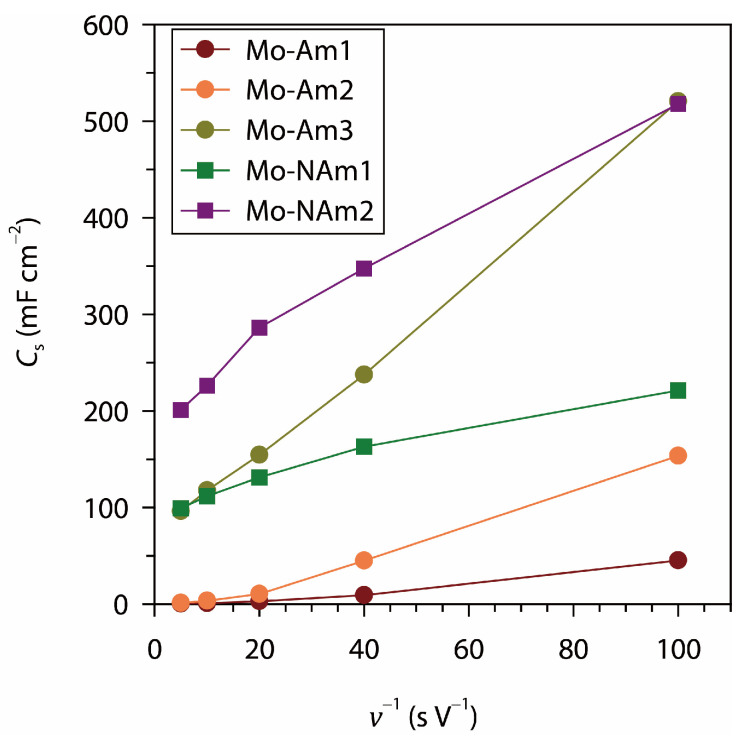
Relationship between areal specific capacitance (*C_s_*) and the reciprocal of CV scan rate (*v*^−1^) for MoN_*x*_ (Mo-Am1, Mo-Am2, Mo-Am3) and MoO_2_ (Mo-NAm1, Mo-NAm2) coatings.

The specific capacitances were calculated based on the above CV, and Figure 8 presents the relationship between *C*_s_ and the reciprocal of the scan rate (*v*^−1^). This plot provides insight into the kinetic limitations and diffusion contribution during charge storage. An ideally pure EDLC system exhibits scan-rate-independent capacitance, while diffusion-controlled faradaic processes show a strong dependence on scan rate, typically leading to an increase in *C*_s_ at lower scan rates. In this context, the nearly invariant *C*_s_ values of Mo-Am1 with respect to *v*^−1^ indicate its EDLC-dominated behavior. Mo-Am2 and Mo-Am3 display a clear linear increase in *C*_s_ with increasing *v*^−1^, indicating the growing contribution of diffusion-controlled redox reactions. This behavior reflects the increased participation of electrochemically active species within the electrode bulk as slower scan rates allow sufficient time for ion diffusion into inner pores and active sites.

The MoO_2_ coatings exhibit significantly higher *C*_s_ values across all scan rates, with Mo-NAm2 showing the steeper slope in the *C*_s_ vs. *v*^−1^ plot than Mo-NAm1. This steep slope signifies a substantial diffusion-controlled contribution while maintaining a considerable capacitive component. With CV rate of 10 mV s^−1^, the *C*_s_ of Mo-Am3 (521 mF cm^−2^) and Mo-NAm2 (518 mF cm^−2^) were similar, but with faster CV scans, the latter exhibited significantly larger capacitances than the former. At 200 mV s^−1^, the *C*_s_ of Mo-NAm2 was evaluated to be 201 mF cm^−2^, as compared with 96 mF cm^−2^ of Mo-Am3. The enhanced performance of Mo-NAm2 can be attributed to its optimized microstructure and substantial electronic conductivity, which promotes efficient ion transport and rapid redox kinetics.

For comparison, the specific capacitances of various molybdenum compounds are compiled in Table 3, from which it can be seen that Mo-Am3 and Mo-NAm2 exhibit moderate capacitive properties. While their capacitance values are relatively lower compared to other Mo-based electrodes, most of which are in the form of nanocomposite structures, it is anticipated that fine-tuning the microstructure of Mo-Am3 and Mo-NAm2 could lead to improved performance.

Taking into account the weight change during the conversion from Mo to MoN*_x_* or MoO_2_, the mass of the active electrode layer was estimated to be 27.3 mg for Mo-Am3 and 31.7 mg for Mo-NAm2 (details in Supplementary Materials). Accordingly, the gravimetric specific capacitances, based on the CV scans at 10 mV s^−1^, were found to be 16.8 F g^−1^ for Mo-Am3 and 14.4 F g^−1^ for Mo-NAm2. The long-term stability of both Mo-Am3 and Mo-NAm2 was examined through extended CV tests at a scan rate of 200 mV s^−1^, which showed that Mo-Am3 and Mo-NAm2 electrodes retained 84% and 81% of their initial capacitance, respectively, after 5000 cycles of charge–discharge.

**Figure 9:**
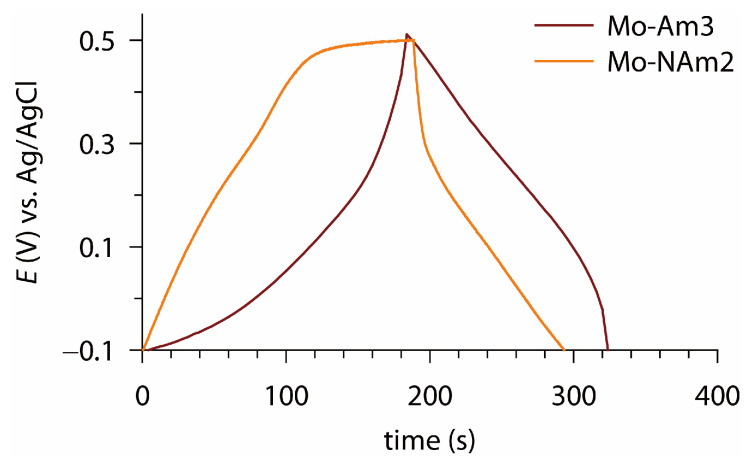
Galvanostatic charge–discharge curves of Mo-Am3 and Mo-NAm2 electrodes measured at a current density of 0.1 mA cm^−2^, and in the potential window of −0.1 to +0.5 V vs. Ag/AgCl.

Figure 9 presents the GCD curves of Mo-Am3 and Mo-NAm2, recorded at a current density of 0.1 mA cm^−2^, which reveal distinct energy storage behaviors for the two electrodes. Mo-Am3 exhibits nearly symmetric triangular charge–discharge curves with minimal IR drop, indicating dominant capacitive behavior and superior electrical conductivity. In contrast, Mo-NAm2 shows noticeable voltage plateaus during charging and a pronounced voltage drop at the initial stage of discharge, suggesting a battery-like faradaic reaction associated with Mo redox transitions. These results indicate that the MoN*_x_*-based electrode is favorable for high-rate capacitive energy storage, while the MoO_2_-based electrode stores charge mainly through diffusion-controlled redox processes.

Similarly to the present work, a recent study by Ren et al. reported the ammonolysis of Mo foil to fabricate MoN supercapacitor electrodes [39]. However, the synthesis condition in that study differs slightly from those in the present work. Specifically, they treated Mo foil in NH_3_ gas at 500 °C for 3 h to form the MoN coating, which is significantly different in both temperature and duration compared to our conditions of 1000 °C for up to 20 h. Moreover, the thickness of the MoN layer formed in their study was not specified, but it is likely that a very thin layer was formed, resulting in a relatively low amount of MoN. In contrast, our study demonstrates the ability to achieve a much higher MoN loading, which represents a key distinction.

## 4. Conclusions

This study demonstrates a straightforward and scalable thermal strategy for producing molybdenum nitride and oxide layers directly on Mo foil, resulting in binder-free and self-supported electrodes with tunable structural and electrochemical properties. Controlled ammonolysis in NH_3_ yielded mixed phases of hexagonal MoN and cubic Mo_2_N, while subsequent treatment in N_2_ facilitated the transformation into MoO_2_, accompanied by microstructural evolution and volumetric expansion. Structural analyses confirmed that increasing nitrogen incorporation and oxidation state systematically influenced electronic structure and surface morphology. Electrochemical evaluation revealed that the charge storage behavior evolves from predominantly electric double-layer capacitance in thin MoN*_x_* layers to hybrid pseudocapacitive and diffusion-controlled mechanisms in thicker MoN*_x_* and MoO_2_ coatings. The MoN*_x_* and MoO_2_ electrode exhibited superior rate performance and achieved areal specific capacitances of ≈520 mF cm^−2^ at 10 mV s^−1^, highlighting the beneficial combination of enhanced conductivity and abundant redox-active sites.

Compared to conventional slurry-based processing methods, the present approach offers significant advantages in terms of interfacial adhesion and mechanical stability. In addition to its favorable electrochemical performance, the MoN*_x_* and MoO_2_ electrodes presented in this work offer several practical advantages for supercapacitor applications. The direct thermal conversion of commercially available Mo foil provides a simple, scalable, and cost-effective fabrication route without the need for polymer binders, conductive additives, or slurry processing. Moreover, the intrinsically high electrical conductivity of MoN*_x_* and MoO_2_, combined with their pseudocapacitive charge storage behavior, enables fast charge–discharge capability and robust rate performance. Overall, this work demonstrates that MoN*_x_* and MoO_2_ layers formed on thermally treated Mo foil constitute a promising platform for high-performance supercapacitor electrodes.

## Figures and Tables

**Table 1:** Samples prepared by heating Mo foil under various conditions, and phase identification ^1^ by synchrotron XRD.

Sample	Heat Treatment	XRD Phase
Mo-Am1	(NH_3_) 1000 °C, 2 h	δ-MoN, γ-Mo_2_N
Mo-Am2	(NH_3_) 1000 °C, 10 h	δ-MoN, γ-Mo_2_N
Mo-Am3	(NH_3_) 1000 °C, 20 h	δ-MoN, γ-Mo_2_N
Mo-N1	(N_2_) 1000 °C, 10 h	Mo_16_N_7_
Mo-N2	(N_2_) 1500 °C, 2 h	MoO_2_
Mo-NAm1	(NH_3_) 1000 °C, 10 h/(N_2_) 1000 °C, 2 h	MoO_2_
Mo-NAm2	(NH_3_) 1000 °C, 20 h/(N_2_) 1000 °C, 2 h	MoO_2_
Mo-O1	(air) 500 °C, 0.5 h	α-MoO_3_

^1^ Based on the ICSD data for δ-MoN (no. 99452), γ-Mo_2_N (no. 172802), MoO_2_ (no. 24322), and MoO_3_ (no. 76651).

**Table 2:** Lattice constants determined by Le Bail fitting of the synchrotron XRD, compared with the literature data for δ-MoN [34], γ-Mo_2_N [33], and MoO2 [64].

Sample	δ-MoN (Hexagonal, *Z* = 8)			γ-Mo_2_N (Cubic, *Z* = 2)	
	*a* (Å)	*c* (Å)	*V* (Å^3^)	*a* (Å)	*V* (Å^3^)
Mo-Am1	5.7412(1)	5.625(1)	160.56(6)	4.16795(5)	72.405(2)
Mo-Am2	5.738(1)	5.622(1)	160.28(6)	4.1708(4)	72.55(2)
Mo-Am3	5.738(2)	5.622(1)	160.29(8)	4.1705(4)	72.54(2)
Literature	5.7356	5.6281	160.34	4.1616	72.07
**Sample**	**MoO_2_ (Monoclinic, ** ***Z* = 4)**				
	***a* (Å)**	***b* (Å)**	***c* (Å)**	**β (°)**	***V*** **(Å^3^)**
Mo-NAm1	5.6119(4)	4.8559(4)	5.6267(4)	120.935	131.52(2)
Mo-NAm2	5.6121(5)	4.8557(6)	5.6274(5)	120.944	131.53(2)
Literature	5.6109	4.8562	5.6285	120.95	131.52

**Table 3:** Brief review of the specific capacitances (*C*_s_) per area (in mF cm^−2^) or per mass (in F g^−1^) of various Mo compounds.

Electrode	*C*_s_	Test Condition	Ref.
MoN/Mo_2_N composite	306.7 F g^−1^	GCD (1 A g^−1^)	[38]
MoN@Mo	10 F cm^−2^	GCD (1 A g^−1^)	[39]
MoN on N-doped carbon cloth	467.6 mF cm^−2^	GCD (5 mA cm^−2^)	[49]
Mo_2_N (dense film)	55 mF cm^−2^	CV (5 mV s^−1^)	[67]
Mo_2_N (nanobelts)	160 F g^−1^	CV (5 mV s^−1^)	[68]
Mo-Am3	521 mF cm^−2^	CV (10 mV s^−1^)	^1^
MoON/TiN	736.6 mF cm^−2^	CV (10 mV s^−1^)	[37]
Mo@MoO_2_	205.1 F g^−1^	GCD (1 A g^−1^)	[69]
MoO_2_/graphene	140 F g^−1^	GCD (1 A g^−1^)	[70]
Mo-NAm2	518 mF cm^−2^	CV (10 mV s^−1^)	^1^

^1^ This work.

## Data Availability

The data presented in this study are available on request from the corresponding author due to privacy.

## Supplementary Materials

The following supporting information can be downloaded at: https://www.mdpi.com/article/10.3390/ma18245649/s1, Figure S1: Synchrotron XRD patterns of Mo-derived coatings; Figure S2: Le Bail fitting of synchrotron XRD pattern for Mo-Am1, Mo-Am2, Mo-Am3, Mo-NAm1, and Mo-NAm2; Figure S3: Relationship between mass-normalized specific capacitance (*C*_s_) and the reciprocal of CV scan rate (*v*^−1^) for MoN*_x_* (Mo-Am1, Mo-Am2, Mo-Am3) and MoO_2_ (Mo-NAm1, Mo-NAm2) coatings; Section S1. Estimating mass of coated MoN*_x_* and MoO_2_ layers on Mo foil.
