# Supplementary material for: Molybdenum Nitride and Oxide Layers Grown on Mo Foil for Supercapacitors
Source: Materials (Basel). 2025 Dec 16;18(24):5649. doi: 10.3390/ma18245649 (PMC12734594; doi:10.3390/ma18245649)
Supplement: Supplementary file 1 [file materials-18-05649-s001.zip › materials-4041720-supplementary.pdf]

Supplementary Materials for

Molybdenum nitride and oxide layers grown on Mo foil for supercapacitors

Dong Hyun Lim, Young-Il Kim*

Department of Chemistry, Yeungnam University, Gyeongsan, Gyeongbuk 38541, Republic of Korea

*Correspondence: yikim@ynu.ac.kr

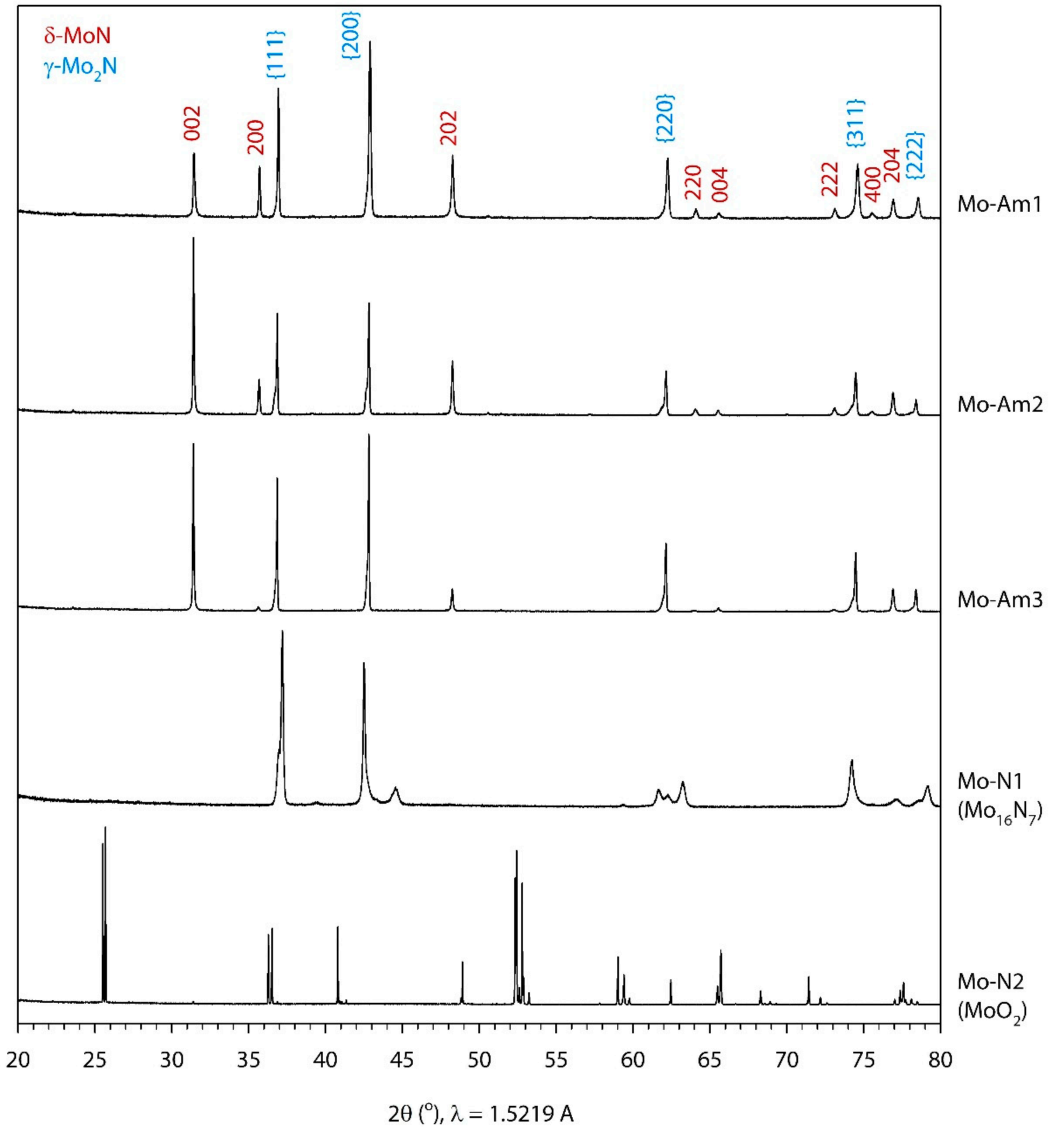

Figure S1. Synchrotron XRD patterns of Mo-derived coatings.

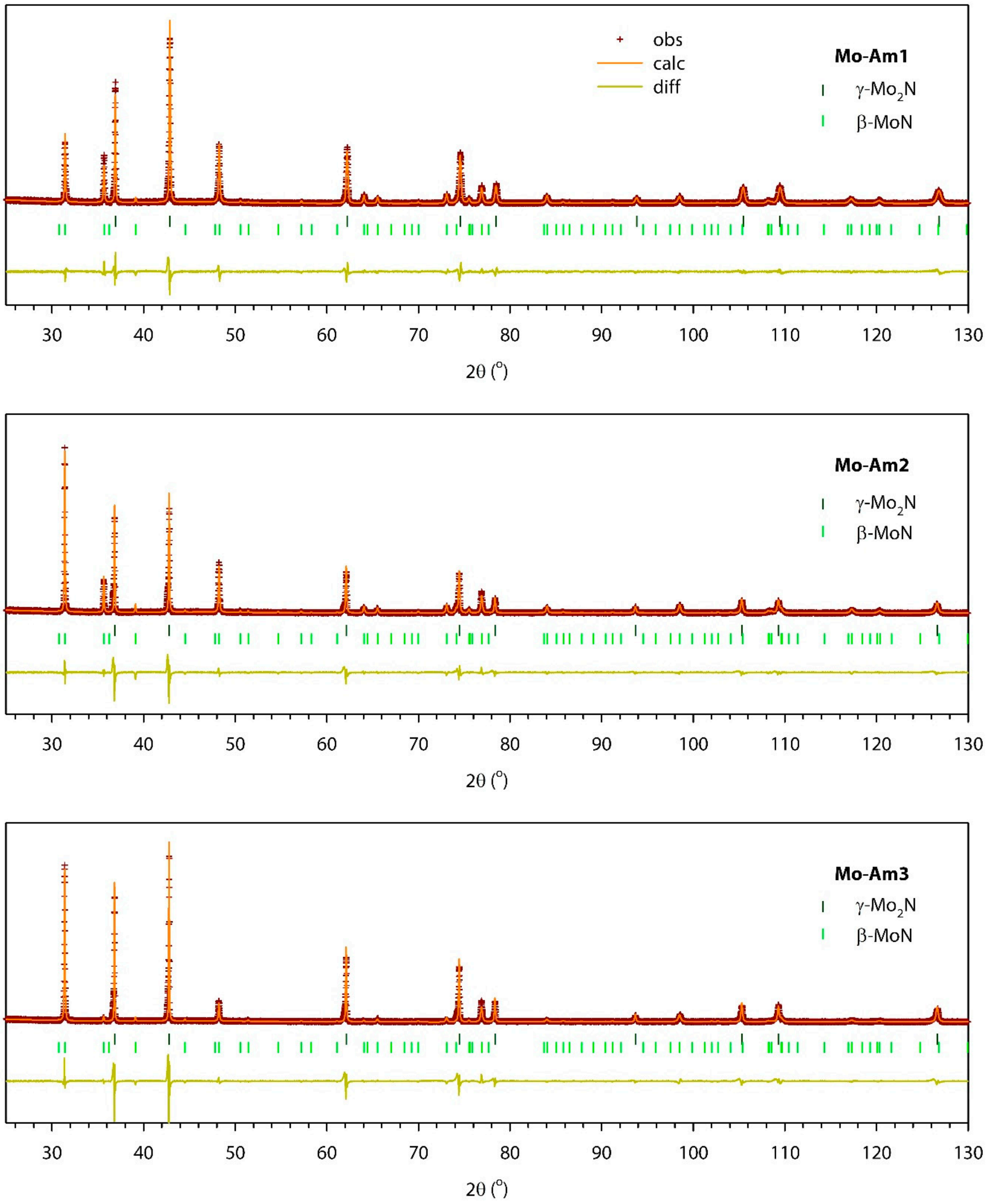

Figure S2 (continued)

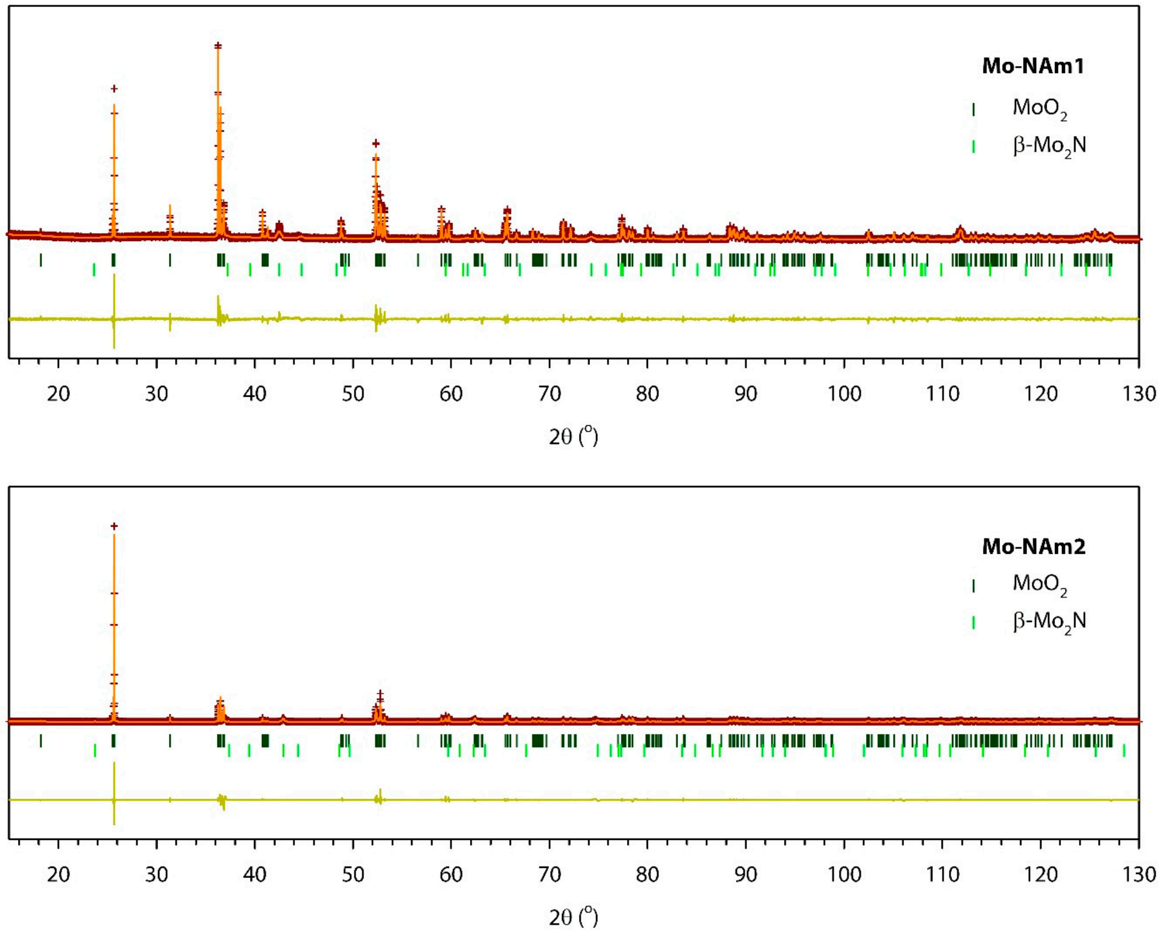

Figure S2. Le Bail fitting of synchrotron XRD pattern for Mo-Am1, Mo-Am2, Mo-Am3, Mo-NAm1, and Mo-NAm2.

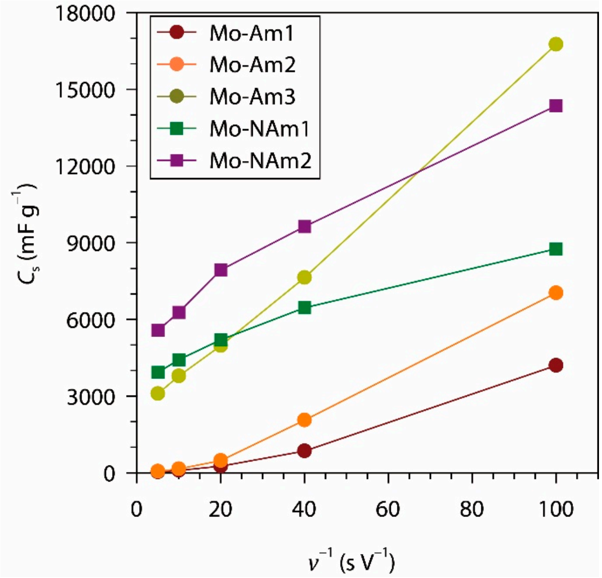

Figure S3. Relationship between mass-normalized specific capacitance (C_s) and the reciprocal of CV scan rate (v^{-1}) for MoN_x (Mo-Am1, Mo-Am2, Mo-Am3) and MoO_2 (Mo-NAm1, Mo-NAm2) coatings.

Section S1. Estimating mass of coated MoN_x and MoO_2 layers on Mo foil

Before and after each of the heat treatments for preparing Mo-Am1, Mo-Am2, Mo-Am3, Mo-NAm1, and Mo-NAm2 samples, the foil specimen was weighed. Using the weight change (Δwt) data, the mass loading for the MoN_x and MoO_2 electrodes, used in the electrochemical analysis, was estimated as below.

S1.1. MoN_x (Mo-Am1, Mo-Am2, Mo-Am3)

weight (g)	① initial	② increment	③ MoN_x	④ active electrode
Mo-Am1	0.3019	0.0043	0.0485	0.00948
Mo-Am2	0.3014	0.0087	0.0981	0.0192
Mo-Am3	0.3040	0.0124	0.1398	0.02734

① Initial weight of Mo foil (1.5×1.5 cm^2)

② Weight gain after a heat treatment in NH_3, which are attributed to the nitridation. Therefore, this increment corresponds to the weight of N in the coated layer.

③ The coated layer consists of MoN and Mo_2N, and we approximate the overall composition to be Mo:N = 3:2. Accordingly, the weight of coated MoN_x is calculated as follows:

$$\text{weight of MoN}_x = \text{weight of N} \times \frac{\text{FW}(\text{Mo}_3\text{N}_2)}{2 \times \text{FW}(\text{N})}$$

④ For the electrochemical analysis, each electrode was prepared to expose only one side of the foil and the area of 0.88 cm^2. Accordingly, the weight of MoN_x serving as the electrode, is calculated as follows:

$$\text{active electrode weight of MoN}_x = \text{total weight of MoN}_x \times \frac{1}{2} \times \frac{0.88}{1.5^2}$$

S1.2. MoO_2 (Mo-NAm1, Mo-NAm2)

weight (g)	① initial	② increment	③ MoO2	④ active electrode
Mo-NAm1	0.3016	0.0142	0.1135	0.02220
Mo-NAm2	0.3003	0.0203	0.1623	0.03174

① Initial weight of Mo foil (1.5×1.5 cm^2)

② Weight gain after a two-step heat treatment in NH_3 and N_2 that results in MoO_2. Therefore, this increment corresponds to the weight of O in the coated layer.

③ The weight of coated MoO_2 is calculated as follows:

$$\text{weight of MoO}_2 = \text{weight of O} \times \frac{\text{FW}(\text{MoO}_2)}{2 \times \text{FW}(\text{O})}$$

④ Similarly to the above (S1.1), the weight of MoO_2 serving as the electrode, is calculated as follows:

$$\text{active electrode weight of MoO}_2 = \text{total weight of MoO}_2 \times \frac{1}{2} \times \frac{0.88}{1.5^2}$$
